# Selected Natural Products in Neuroprotective Strategies for Alzheimer’s Disease—A Non-Systematic Review

**DOI:** 10.3390/ijms23031212

**Published:** 2022-01-21

**Authors:** Karolina Wojtunik-Kulesza, Tomasz Oniszczuk, Jarosław Mołdoch, Iwona Kowalska, Jarosław Szponar, Anna Oniszczuk

**Affiliations:** 1 Department of Inorganic Chemistry, Medical University of Lublin, Chodźki 4a, 20-093 Lublin, Poland; 2 Department of Thermal Technology and Food Process Engineering, University of Life Sciences in Lublin, Głęboka 31, 20-612 Lublin, Poland; tomasz.oniszczuk@umlub.pl; 3 Department of Biochemistry and Crop Quality, Institute of Soil Science and Plant Cultivation, State Research Institute, 24-100 Puławy, Poland; jmoldoch@iung.pulawy.pl (J.M.); ikowalska@iung.pulawy.pl (I.K.); 4 Toxicology Clinic, Clinical Department of Toxicology and Cardiology, Medical University of Lublin, Stefan Wyszyński Regional Specialist Hospital, Al. Kraśnicka 100, 20-718 Lublin, Poland; szponar.jarek@gmail.com

**Keywords:** Alzheimer’s disease, neurodegeneration, dementia, polyphenols, terpenes, secondary plant metabolites

## Abstract

Neurodegenerative disorders such as Alzheimer’s disease (AD) are distinguished by the irreversible degeneration of central nervous system function and structure. AD is characterized by several different neuropathologies—among others, it interferes with neuropsychiatrical controls and cognitive functions. This disease is the number one neurodegenerative disorder; however, its treatment options are few and, unfortunately, ineffective. In the new strategies devised for AD prevention and treatment, the application of plant-based natural products is especially popular due to lesser side effects associated with their taking. Moreover, their neuroprotective activities target different pathological mechanisms. The current review presents the anti-AD properties of several natural plant substances. The paper throws light on products under in vitro and in vivo trials and compiles information on their mechanism of actions. Knowledge of the properties of such plant compounds and their combinations will surely lead to discovering new potent medicines for the treatment of AD with lesser side effects than the currently available pharmacological proceedings.

## 1. Introduction

Alzheimer’s disease (AD) is the most common cause of dementia globally. According to the World Alzheimer Report 2019, over 50 million patients suffer from AD. Long-lasting studies have revealed the multi-factorial character of the diseases that contribute to the complexity of the disorder [1].

Among the most intensively studied pro-neurodegenerative factors are the following: oxidative stress, amyloid-β accumulation leading to senile plaques, low level of neurotransmitters in the brain (i.e., acetyl- and butyrylcholine), high level of metal ions in the organism, and too high activity of monoamine oxidase (MAO) and neuroinflammation [2,3,4]. An effective substance should therefore be active against several possible pro-degeneration factors.

Similarly to other disorders, in the case of neurodegeneration, quick diagnosis of disease is crucial for effective treatment. Hence, an additional complication results from the non-specific character of the first AD symptoms. In the first stage of AD development, symptoms are similar to typical chronic tiredness, along with memory disturbances. Unfortunately, this stage is presented as crucial for treatment success. The middle stage and later symptoms are more characteristic for neurodegeneration. In this case, significant memory and learning disturbances are apparent, along with insomnia and problems with interpersonal contacts, which lead to complete dependency on third parties [1].

In the search for treatment for AD, besides synthetic drugs, recently, substances of natural origin have been very popular. It is known that plants are inexhaustible source of active compounds that are used as drugs in numerous disorders. Among the secondary plant metabolites, polyphenols and terpenes are the most popular due to their rich biological activities such as antioxidant, sedative, anti-inflammatory, antibacterial, enzyme inhibitory effects [5].

The presented review is focused on natural compounds that reveal positive activity (in vitro, in silico, in vivo) against various Alzheimer’s disease factors, along with their mechanisms of action.

## 2. Activity Targeting Cholinergic Neurotransmission

The cholinergic system is associated with a number of cognitive functions, e.g., learning and memory. Cholinergic neurons are major source of innervation in the cortex and hippocampus. They release choline O-acetyltransferase (ChAT), which takes part in the production of acetylcholine (ACh) by catalyzing the transfer of acetyl group from the coenzyme acetyl-CoA to choline [2]. Acetylcholinesterase (AChE) and butyrylcholinesterase (BuChE), expressed at lower levels than AChE), hydrolyze ACh back to choline [6]. The cholinergic hypothesis proposes that the decline of cholinergic neurotransmission and loss of these neurons in AD patients cause cognitive deficits [2]. Researchers found that besides the decrease in the mRNA expression level of ChAT in the AD brain, its activity is also reduced, which is asynchronous with synaptic loss [7].

For many years, research on AChE inhibitors has been a major avenue for drug development for AD. In fact, three out of the four anti-AD drugs approved by the Food and Drug Administration are AChE inhibitors. Unfortunately, the results of the clinical studies demonstrate that their therapeutic effects are not as effective as expected. The brain of AD patients contains very high concentrations of BuChE [8]. Therefore, other anti-AD strategies targeting cholinergic neurons include BuChE inhibition and promotion of ChAT expression, as well as protection of cholinergic neurons by stimulating the expression of nerve growth factor (NGF), brain-derived neurotrophic factor (BDNF), and their receptors [6].

Both AChE and BuChE are associated with aggregation of Aβ plaques. AChE is able to increase Aβ peptide fibril aggregation to form Aβ–AChE complexes. In general, AChE activity is decreased in the AD brain, but its concentration could be enhanced while binding to Aβ plaques. However, the association between AChE and BuChE with other AD hallmarks remains largely unexplored [9].

An in vitro study on aqueous extracts from 80 traditional Chinese medicinal plants (from families drawn from Berberidaceae, Ranunculaceae, and Rutaceae) showed that extracts rich in isoquinoline alkaloids effectively inhibit AChE activity [10]. Moreover, extracts of *Berberis bealei, Coptis chinensis*, and *Phellodendron chinense*, which are characterized by a high content of isoquinoline alkaloids, were found to substantially constrain AChE. Furthermore, combinations of three of the alkaloids palmatine, berberine, and coptisine demonstrate a synergistic enhancement of ACh restriction. It is likely that the way of AChE inhibition by crude extracts of *Coptis chinensis*, *Berberis bealei*, and *Phellodendron chinense* is due to of this synergism of alkaloids. It should be emphasized that none of the active extracts are cytotoxic at the concentrations that limit AChE [11].

It was also observed many years ago that ‘Compound Danshen Tablet’, a traditional Chinese medicine, can improve spatial cognition. However, the in vivo neuroprotective mechanism of the Compound Danshen Tablet in models of spatial memory impairment in mice was not evaluated until 2014. The results of the research conducted by Teng et al. [12] have found that this medicine increased ChAT expression in the brain, induced BDNF production, and activated the protein kinase C (PKC) receptor to improve spatial recognition in an AD rat model.

Besides Compound Danshen Tablet, various other preparations and extracts used in Chinese medicine have demonstrated therapeutic effects on AD through their effects on the expression of NGF, BDNF, and their related receptors in vivo. The very popular Bushen-Yizhi formula was noted, for example, to be able to regulate NGF signal transduction and the anti-apoptotic cholinergic pathway to improve memory impairment in an AD rat model [13]. Moreover, bioactive components of ginger 6-shogaol have increased the levels of NGF and improved scopolamine-induced memory impairment in animal models of dementia [14]. In addition, *Xanthoceras sorbifolium* extracts, rich in transhinone II, were seen to save dendritic spines through the BDNF signal transduction pathway and improve cognition in an AD rat model. Moreover, tanshinone IIA was found to be helpful in promoting depolarization-induced BDNF synthesis [15], and Polygonum multiflorum Thunberg complex was recognized to increase BDNF level and synapse number in the hippocampus of an AD mice model [16]. Finally, extracts from *Huperzia serrata* (studies on AD mice) were found to inhibit AChE activity and ameliorate the cognitive impairment [17].

Beyond the aforementioned substances, extracts from *Crocus sativus* in in vitro study showed moderate inhibitory activity against AChE, while crocetin, dimethylcrocetin, and safranal extracted from *C. sativus* have all been found to possess moderate AChE inhibitory activities (IC_50_ values below or around 100 µM). Accordingly, results of kinetic analysis exhibited mixed-type inhibition. This was verified by in silico docking studies. Here, safranal was found to interact only with the binding site of the AChE, but crocetin and dimethylcrocetin bound simultaneously to the catalytic and peripheral anionic sites. The presented findings confirm previous results about the beneficial action of saffron against AD and may be of value for the development of novel therapeutic agents based on carotenoid-based dual binding inhibitors [18].

Other plant metabolites and extracts with anti-AD potential are listed in Table 1.

## 3. BACE-1 Inhibitory Activity

One of the most studied neurodegenerative factors is amyloid-β accumulation. This plays a key role in the aggregation of neurotic senile plaques. In young healthy organisms, the Aβ peptides are released outside of cells, removed, and excreted. With age, the process is disturbed, and the ability of the organism to get rid of amyloid compounds is reduced. This leads to accumulation of the forms in the brain. Many years of research have allowed for detailed analysis of the creation of Aβ structures, as well as their influence on brain functioning.

It is known that amyloid-β is created in two stages. In the first, the amyloid precursor protein (APP) cleaves the enzyme BACE-1 (β-secretase), known also as Asp2. This stage leads to the generation of soluble versions of protein (sAPPβ) and a 99 amino acid fragment (C99), which, in turn, is treated with a second enzyme γ-secreatse to produce peptides composed of 38–43 amino acids (termed epsilon, ε; zeta, ζ; and gamma, γ) and AICD (APP intracellular domain) [31]. Among these, the greatest threat is Aβ40 and Aβ42, both being main products and simultaneously playing crucial roles in senile plaque creation. It is worth stating that Aβ42 is the most toxic form and has high predispositions towards aggregating. This is particularly evident in a familial form of AD [32,33].

The importance of BACE-1 activity results from its ability to determine total amyloid-β production and to induce overproduction of toxic Aβ42. In the luminal surface, it is a membrane anchored aspartyl protease responsible for APP cleaving [33]. From a structural point of view, the enzyme is characterized by a large and elongated active site (1000 Å and 20 Å, respectively), along with a large catalytic domain with centrally located catalytic aspartates Asp32 and Asp228 [34]. Molecular simulations revealed that the key interacting residue is Arg307 at the edge of the catalytic cleft [35]. Additionally, molecular simulations revealed four residues creating group-specification in the ligand binding side, namely, Pro70, Ile110, Ile126, and Asn233 [36]. It is known that the catalytic activity of β-secretase involves aspartic proteases hydrolyzing peptide bonds.

In accordance with Shimizu et al. [37], BACE-1 activity is directly affected by the behaviors of water (Wat) molecules, and the molecules participate in the creation of the following hydrogen-bonding network: Wat–Ser35–Asp32–Wat–Asp228. An equally important amino acid residue is Leu30, which is responsible for stabilizing the bound inhibitor conformation [37].

Several inhibitors that were tested in phase II or phase III clinical trials were the result of multiple years of research on the enzyme. Among them are the following: lanabecestat, verubecestat, elenbecestat, atabecestat, and umibecestat [31]. Selected doses of the substances led to reduction of Aβ in the cerebrospinal fluid by 90%. Of note, in the clinical trials, a high level of BACE inhibition to achieve Aβ lowering was the goal, and the effects of lower doses of active substances were not investigated [38].

Information obtained from the studies indicates that the optimal therapeutic window might be before the onset of appreciable Aβ plaque deposition; hence, the solution can be used in preventive therapy [39]. It is known that the effectiveness of preventive therapy depends on the length of drug administration, and thus side effects of the solution must be minimalized. In the case of BACE-1 inhibitors, adverse effects were observed both in non-clinical studies and clinical trials. The following negative changes were observed: off-target effects: retinal toxicity in an animal model and hepatotoxicity in humans [38,40], and mechanism-based effects: cognitive decline, anxiety, weight loss, sleep disturbances, and suicidal ideation [41,42,43].

When therapy based on BACE inhibitors is considered, a milestone is appropriate dosage. In accordance with available study results, there is a therapeutic window for the enzyme inhibitor concentration, namely, 3 nM concentration of BACE inhibitor caused 40% inhibition of APP processing, whereas concentration > 300 nM led to significant inhibition of BACE-dependent neuronal growth cone collapse [38,44].

In this subsection, attention will be focused on natural compounds derived from plants that were studied in vitro, in silico, and in vivo towards their BACE-1 inhibitory activity (Table 2). Taking into account an inexhaustible source of natural active compounds, it is highly probable to find substances that are highly active inhibitors that come without adverse side effects.

## 4. α-Synuclein Inhibition

Research results provide scientific evidence confirming the correlation of neuronal mitochondrial dysfunction with the pathogenesis of neurodegenerative diseases, including AD [62]. Abnormal accumulation of α-synuclein induces an alteration of normal mitochondrial function, leading to neuronal degeneration and strong oxidative stress [63]. This low molecular weight protein can activate microglia and release proinflammatory cytokines such as NO and ROS, resulting in microglial activation, neuronal death, and further inflammation [64].

Synucleinopathic disorders involve the accumulation of inclusions rich in α-sunuclein [65,66]. Different types of aggregates, e.g., fibrils, protofibrils, and oligomers, are produced during aggregation of these protein in synucleinopathies. Some aggregated species might be neurotoxic and lead to neurodegeneration [67]. For this reason, targeting neuronal accumulation of α-synuclein is appealing as a promising approach to delaying the progression of AD [68,69].

Limited amounts of research have been published thus far on inhibiting α-synuclein aggregation by natural compounds [70]. Ehrnhoefer et al. [71], however, demonstrated that epigallocatechin-3-gallate (EGCG) inhibits the fibrillogenesis of these protein by directly binding to natively unfolded polypeptides and preventing their conversion into toxic aggregation intermediates. Moreover, computational molecular docking analysis showed that this plant compound preferentially bound the *C*-terminus of α-synuclein. Other studies have revealed that epigallocatechin-3-gallate promotes the production of unstructured, nontoxic α-synuclein. These phenomena suggest its favorable effect on aggregation pathways in AD. In addition, the results of studies conducted by Hornedo-Ortega et al. [72] showed that protocatechuic acid (doses 10, 20, 50, and 100 μmol/L) inhibits Aβ and α-synuclein aggregation. What is more, protocatechuic acid disturbs the stability of prefabricated fibrils and inhibits Aβ- and α-synuclein-induced PC12 cell death.

## 5. MAO Inhibition

Monoamine oxidase (MAO) is an enzyme bound with the mitochondria that catalyzes the oxidative deamination of a range of neurotransmitters e.g., serotonin, tyramine, norepinephrine, and dopamine. This process produces (during the biochemical reaction) several harmful side compounds, including peroxides, ammonia, and aldehydes. This enzyme occurs in MAO-A and MAO-B isoforms. They show remarkable sequence similarity but differ in their substrate-inhibitor recognition sites and presence within the tissues. MAOs catalyze the oxidative deamination of several monoamines and play important roles in metabolism-released neurotransmitters [73]. Both isoforms MAO-A and MAO-B possess 73% sequence similarity; however, in the central nervous system, the MAO-A form is present mostly in catecholaminergic neurons, whereas the MAO-B form is mostly found in serotonergic neurons and in the glia [74].

In many neurodegenerative diseases, including AD, amended levels of neurotransmitters are observed [75]. Activated MAO causes amyloid beta aggregation by two successive cleft b-secretase and g-secretase effects upon the amyloid precursor protein. Moreover, this enzyme participates in cognitive damage through the destruction of cholinergic neurons, as well as through disturbance of the cholinergic system. MAOs also regulate mood control, motor function, and brain and motivational functions [76,77,78]. MAO enzyme inhibition causes an anti-AD effect as a result of oxidative stress decrease prompted by MAO. Inhibitors of MAO can block the catalytic activity of the enzyme and slow down the catabolism process of various monoamines. They also increase the production of the monoamine neurotransmitters that are accumulated in the nerve terminals. Inhibitors of this enzyme are applied as medicines in diseases where MAO is over-expressed. These drugs halt the production of neurotoxic side substances and thus prevent neuronal damage [79].

Radioenzymatic screening in brain autopsy has revealed that the alterations in MAO-A and MAO-B in the prefrontal cortex are present from the beginning of AD and remain constant in the later AD stages. In addition, levels of MAO-A and MAO-B and/or mRNA may rise in various brain areas, including in the frontal lobe of the neocortex and also in the parietal, temporal, occipital, and frontal cortices [73]. Studies using immunostaining demonstrate that in AD, the MAO-B level is significantly increased in the hippocampus and in the cortical areas, whereas MAO-A activity is enhanced in the frontal pole and hypothalamus [80]. This indicates that cell loss and substantial gliosis in these brain areas has occurred. The presence of MAO-A in the neurons is implicated in the pathology of AD as a predisposing factor, and activation of MAO occurs during AD cognitive dysfunction.

Monoamine neurotransmitter systems play significant roles in cognition at the biomolecular level, especially in memory, attention, paranoid thinking, behavior, and emotion, as well as orientation [73,81]. Oxidative stress related to MAO is a well-recognized cause of neurotransmitter dysfunction in AD [82]. Indeed, neuroinflammation participates significantly in cognitive loss and in oxidative stress, and in AD, MAO may have pro-inflammatory effects, as activated MAO increases levels of monoamine in the brain. Moreover, research indicates that MAO alters other neurotransmitter systems resulting in cogitative impairments [83]. In addition, changes in the concentration of dopamine and serotonin acid metabolites (homovanillic acid and 5-hydroxyindole-3-acetic acid), mediated by MAO and established via AD-related mouse models pathology, are known to be related to cognitive deficits [84].

Amyloid plaques are also produced through MAO activation. High oxidative stress in AD patients results in amyloid plaque formation, and an increased level (>3-fold) was notably found in sensitive astrocytes around plaques (amyloid-beta). In astrocytes, this increasing level of MAO-B is hypothesized to result in excessive deamination of monoamines and the release of large amounts of oxygen radicals; hence, it could contribute to the progress of AD. Research on AD mice demonstrates that MAO-B is firmly related with the formation of GABA (gamma-aminobutyric acid) in sensitive astrocytes, and this effect brings about memory deterioration [85].

Many natural substances are used in medicine, such as MAO-A and MAO-B inhibitors, in the treatment of neurodegenerative diseases, including AD. MAO-B inhibitors not only enhance dopaminergic neurotransmission, but they also reduce the radical production from toxins. Larit et al. [86] isolated quercetin and myricetin from *Hypericum afrum*, as well as genistein and chrysin from *Cytisus villosus*, and evaluated their effect upon recombinant hMAO-A and hMAO-B in in vitro studies. Therein, quercetin, myricetin, and chrysin induced MAO-A inhibition activity with IC_50_ values of 9.93, 1.52, and 0.25 µM, respectively, whereas genistein was found to be a most effective potent inhibitor of MAO-B, with an IC_50_ value of 0.65 µM. In addition, computational docking and dynamic simulation showed its ability to effectuate neuroprotection and MAO-A and MAO-B binding affinity at the molecular level [86].

Baek et al. [87] obtained bisdemethoxycurcumin and demethoxycurcumin from *Curcuma longa*. These compounds were tested for MAO-A and -B inhibitory activity. Both compounds were found to be potential inhibitors against the MAO-B enzyme, with high IC_50_ values [87]. This study suggests that the investigated curcumin derivatives could be potent inhibitors for the treatment of MAO related disorders. Other research [88] isolated alternariol monomethyl ether (AME) from *Alternaria brassicae*. In these experiments, AME exhibited high and selective hMAO-A inhibition. However, this compound was found to be less effective for MAO-B inhibition. Chaurasiya et al. [89], in turn, isolated acacetin 7-methyl ether from *Turnera diffusa* and analyzed its in vitro inhibitory activity against recombinant hMAO-A and hMAO-B. This compound was discovered to be a potent selective MAO-B inhibitor, with an IC_50_ value of 198 nM. Furthermore, the molecular docking and molecular dynamic experiments showed that the compound displayed selectivity stable and strong inhibition of the MAO-B enzyme.

Mohamed et al. [90] isolated 14 compounds from *Zanthoxylum flavum* stems and evaluated their recombinant human MAO inhibition. The results of the study revealed that compound 3-sesamin exhibited potent selective MAO-B impediment (IC_50_ value of 1.45 µM). The promising MAO-B inhibitory activity of sesamin inclined the authors to explore its kinetic properties, binding mode, and the mechanism of MAO-B restriction. Detailed investigation substantiated reversible binding and mixed MAO-B catalytic function constraint by sesamin. This study provided promising findings for further in vivo investigation to confirm the therapeutic potential of sesamin.

Rauhamäki et al. [91] designed and synthesized numerous 3-phenylcoumarin derivatives and subsequently screened them for MAO-B inhibitory activity. They found 24 coumarin derivatives that are promising inhibitors of selected MAO-B (IC_50_ in the range 100 nM–1 µM). This study also researched the best ligand lipophilicity efficiency. This work indicates that in the future, 3-phenylcoumarin derivative drug development can be developed into being pharmacologically more active inhibitors. Yang et al. [92], in turn, synthesized and studied the in vitro activity of 3-arylcoumarin derivatives. In the work, most of these substances exhibited good activity against MAO, AChE, and BuChE enzymes; thus, potentially, they could become anti-AD drugs. The optimal compound was particularly noted to be a very effective inhibitor of MAO (IC_50_ = 27.03 µM).

Other studies have synthesized new derivatives of hydroxypyridinone-coumarin and developed their potential towards AD. These compounds display potential MAO-B inhibitory activity as demonstrated under in vitro assay. Herein, 1-((7-((3-fluorobenzyl)oxy)-2-oxo-2H-chromen-3-yl) methyl)-3-hydroxy-2-methylpyridin-4(1H)-one hydrochloride exhibited the highest active anti-MAO-B (IC_50_ = 14.7 nM). Molecular docking shows that this compound is a potential drug for AD treating because it can bind both substrate and entrance cavity of MAO-B [93].

To discover different inhibitors, Xie et al. developed novel coumarin-dithiocarbamate derivatives. They explored the options for treating AD by restriction of selective MAO-B isoforms. Compound 3-((3-chloro-4-methyl-2-oxo-2H-chromen-7-yl)oxy)propyl2,6-dimethyl-piperidine-1 carbodithioate evidenced the strongest MAO-B (IC_50_ = 0.101 µM). It also did not show any critical toxicity in mice in therapeutic doses, and, according to the researchers, prevented cognitive dysfunction in the AD-infected mice [94]. Repsold et al. [95] produced multitargeted directed ligands based on a coumarin scaffold that demonstrated inhibitory activities at two main enzymes (MAO-B and AChE) for the treatment of AD. Biological assay indicated that one of the coumarin-morpholine ether conjugates was a most promising hMAO-B inhibitor, while one of the coumarin-piperidine conjugates was an effective AChE inhibitor.

Overall, a significant number of structurally distinct synthetic and natural compounds can inhibit both MAO-A and MAO-B isoforms with different degree of potency and selectivity. However, it is not easy to categorize the chemical structures for their affinity towards the MAO-A and MAO-B isoform.

## 6. Anti NFTs Accumulation

The tau proteins play an important role in cell integrity. They are predominantly found in neurons, but small amounts of tau are also located in the astrocytes and oligodendrocytes. The different tau isoforms are encoded by the microtubule-associated gene on human chromosome 17q21 [6]. The microtubule-associated protein tau is disordered and shows high flexibility and lack of a stable conformation. The major hallmark of the tau hypothesis of AD pathogenesis is the formation of NFTs, which are aggregates of abnormal tau proteins. In AD patients, the density of NFTs is related to the degree of cognitive deficit [96], and therefore, tau needs to be detached from microtubules and then transferred into abnormal aggregates before a patient develops AD. This modification is probably caused by a series of post-translational processes, e.g., phosphorylation, glycosylation, nitration, acetylation, ubiquitination, and methylation. Abnormal phosphorylation is the most important modification. In AD, both the total and phosphorylated tau levels increase, along with the disease progression. It has been revealed that tau is 3–4 times more phosphorylated in the brains of AD patients compared to healthy brains [97].

Tau protein hyperphosphorylation causes the dissociation of tau from microtubules and induces abnormal tau aggregation. Approaches to blocking tau-mediated neurotoxicity includes primarily restricting tau post-translational modifications and directly inhibiting tau aggregation. Tau protein dephosphorylation is mainly brought about by protein phosphatase 2A (PP2A). This phosphatase has reduced activity in the AD brain and is a difficult target for drugs [98]. Moreover, research suggests that inhibition of PP2A could induce tau hyperphosphorylation [6]. This fact indicates that PP2A might regulate normal tau protein phosphorylation by preventing excessive activation of tau kinases. It is believed that tau phosphorylation could be the result of equilibrium between tau protein phosphatases and kinases. Therefore, protein kinase inhibitors are usually targeted towards other kinases (rather than directly on PP2A) to impede tau hyperphosphorylation or reduce tau aggregation [99]. When this enzymatic equilibrium is disturbed, tau hyperphosphorylation occurs. This process will lead to NFT formation and cognitive deficits.

In AD animal models, some plant compounds and products have been shown to constrain tau hyperphosphorylation through modulating the activity of glycogen synthase kinase-3 (GSK3) or cyclin-dependent kinase-5 (CDK5), or directly through PP2A control [100]. ‘Tongmai Yizhi’, a decoction derived from Chinese medicine, has been demonstrated to significantly decrease CDK5 and CDK5 expression in the hippocampus of model rats [101]. This decoction contains plants such as *Daemonorops draco* (Willd.), *Panax ginseng* C.A. Meyer, *Rehmannia glutinosa* Libosch, *Alpinia oxyphylla* Miq., *Gastrodia elata* Blume, and *Whitmania pigra* Whitman. Multidrug compatibility is regarded as the essence of Tongmai Yizhi decoction activity. However, due to the complex components and numerous targets involved, fully elucidating its mechanism is challenging [102].

Safflower yellow is one of the traditional Chinese medicines extracted from safflower (*Carthamus tinctorius*), which is suggested to have therapeutic potential for neurodegenerative disorders. Data obtained by Ma et al. [103] indicate that this extract can serve as a therapeutic candidate for AD. They have found that safflower yellow inhibits the GSK-3 activation and GSK-5 signaling pathways so as to protect against tau hyperphosphorylation by Aβ1–42, and in this way improves learning and memory functions in AD model rats.

It is known that ginsenoside Rd (Rd), one of the main active ingredients in *Panax ginseng*, increases PP2A activity and decreases okadaic acid-induced neurotoxicity, as well as tau hyperphosphorylation in vitro and in vivo [104]. Zhang et al. [105] investigated whether Rd could reduce tau phosphorylation and sequential cognition impairment after ischemic stroke. The results of the study demonstrated that Rd treatment reduces ischemia-induced enhancement of tau phosphorylation and ameliorated behavior impairment. Moreover, Rd inhibits the activity of GSK-3β but enhances the activity of protein kinase B (PKB/AKT), an important kinase suppressing GSK-3β activity. The authors also concluded that LY294002, an antagonist for the phosphatidylinositol 3-kinase (PI3K)/AKT signaling pathway, significantly decreases the inhibitory effect of Rd on GSK-3β activity. These findings provide evidence that Rd may reduce cerebral ischemia-induced tau phosphorylation via the PI3K/AKT/GSK-3β pathway.

Epigallocatechin-3-gallate is an active plant metabolite that has therapeutic potential against various disorders, including inhibition of tau aggregation. EGCG interacts with full-length tau protein at several residues with unstable interactions. This compound restricts aggregation of tau and dissolves tau fibrils and oligomers. It is likely that EGCG forms higher-order structures and degrades them without allowing the formation of mature aggregates [106].

Many of the tau aggregation inhibitors are natural plant compounds with antioxidant activity. Crocin from *Crocus sativus* can interfere with tau protein nucleation and inhibit tau protein filament formation in vitro [100]. In vitro, the aqueous extract of *Glycyrrhiza inflata* can improve the growth of the repeat domain and axons in mutant tau protein to prevent tau aggregation. This extract is also able to upregulate unfolded protein response-mediated chaperones to reduce tau misfolding. Cornel iridoid glycoside is the main compound extracted from *Cornus officinalis*. The findings obtained by Yang et al. [107] suggest that it may be used as a promising anti-AD drug. The results presented by the authors provide novel insights into how cornel iridoid glycoside constrains tau hyperphosphorylation. Among other approaches, this compound impedes GSK-3β activity through promoting the phosphatidylinositol 3-kinase/AKT signaling pathway. Moreover, it can elevate PP2A activity via constraining PME-1-induced PP2A catalytic C subunit demethylation, and, subsequently, limiting GSK-3β activity. In this way, cornel iridoid glycoside regulates the crosstalk between GSK-3β and PP2A signaling and, consequently, inhibits tau hyperphosphorylation.

Sonawane et al. [108] screened the potency of baicalein, a polyphenol from the *Scutellaria baicalensis* Georgi, against in vitro tau aggregation and tau filaments dissolution. Their study suggests the potency of baicalein against two pathological tau activities, namely, this plant metabolite efficiently inhibits tau formation by promoting off pathway oligomers, as well as by dissolving tau filaments. This research highlights the potential of baicalein in ameliorating multifactorial neurodegenerative pathologies.

Curcumin works in a similar way as baicalein. Studies indicate that curcumin inhibits the oligomerization of tau and could disaggregate tau filaments [109]. Another plant compound able to constrain tau protein aggregation is resveratrol, which inhibits the aggregation of the repeat domain of tau (and shows several other neuroprotective mechanisms) [110]. Two more are folic acid, which slows down tau aggregation via stabilizing its native state [111], and purpurin, which counteracts tau fibrillization and breaks down the pre-formed fibrils [112].

## 7. Neuroinflammation

A significant factor attributable to neurodegeneration is neuroinflammation. This is a cellular and biochemical response that increases inflammatory mediators (cytokines, chemokines) and activates glia cells and leukocyte invasion of brain tissue [113]. A significant side effect of the process is increased permeability of the BBB (blood–brain barrier). It is known that this neuroinflammation is strictly connected with innate (the first line of defense) and adaptive immune responses. In the case of Alzheimer’s disease, neuroinflammatory contribution to pathogenesis equals that of senile plaques and NFTs [114]. The following neuroinflammatory landscapes that are associated with AD are the most intensive studied:

- Microglia: the resident phagocytes of central nervous system. In the case of AD, the structure binds to soluble Aβ oligomers and Aβ fibrils via the following receptors: SCARA1, CD36, CD14, α6β1 integrin, CD47, and Toll-like receptors. Binding of Aβ with CD36, TLR4, and TLR6 leads to activation of microglia and the production of proinflammatory cytokines and chemokines [114,115].
- Astroglia: accumulates around senile plaques. The structures release cytokines, interleukins, nitric oxide, and other potentially cytotoxic molecules. ApoE is needed for astrocyte-mediated clearance of Aβ, and astrocyte-dependent lipidation of ApoE increases the capability of microglia to clear Aβ [114].

There are many factors that contribute to neuroinflammation. Besides typical pro-neuroinflammatory factors such as senile plaques, there are numerous linkage with phenomena such as (1) systematic inflammation, for which studies revealed explicit correlation with inflammatory changes in the brain [116]; (2) obesity, which is characterized by white fat having a high level of activated macrophages that constantly secrete proinflammatory cytokines [117]; (3) traumatic brain injury leading to microglia activation, which can persist for months or years after traumatic brain injury [118]; and (4) locus coeruleus degeneration being strictly connected with loss of noradrenaline, which is due to compromised microglial migration and Aβ phagocytosis [119].

One of the most often administered tests towards determining anti-inflammatory activity is the carrageenan-induced rat paw edema test. This was applied for assessing the activity of extracts of *Acalypha hispida* (Euphorbiaceae) leaves, which are rich in ellagic acid, gallic acid, and rutin [120]. The basis of the test is a marked edema formation that is mediated by histamine, serotonin, and bradykinins (first phase), as well as the release of prostaglandins and nitric oxide (second phase). The study results revealed that extracts of *Acalypha hispida* significantly decreased edema formation and histamine-induced rat paw edema. The most probable mechanism is based on antihistaminic activity, but inhibition of carrageenan-induced inflammatory responses was also noted; hence, the main components of the studied extract might follow several inflammatory pathways. The compound that is likely to be responsible for the activity is ellagic acid, which is known to constrain COX-2 and NO synthase expression [121].

A well-known and commonly administered secondary plant metabolite is lycopene, a hydrocarbon carotenoid. The compound has demonstrated a variety of biological activities, including high antioxidant activity. In rat model studies, lycopene and human amniotic epithelial cells (HAECs) were used as therapeutic agents for assessing immunomodulatory effects at the choroid plexus. Here, the results revealed that lycopene administration has a significant impact on the level of proinflammatory mediators such as TNF-α and IL-1β. In addition, the metabolite was found to increase the anti-inflammatory mediators IL-10 and TGF-β1 in the cerebro-spinal fluid and hippocampal tissues. Additional analysis revealed that lycopene can positively affect upregulation of Toll-like receptor 4, leading to reversion of Aβ [122].

Terpenes are another group of secondary plant metabolites revealing rich biological activity. The group consists of over 55,000 compounds that are well diversified in terms of structure and effect. Among them are triterpenoids that demonstrate anti-neuroinflammatory activity. For example, interesting results were obtained for novel triterpenoids derived from seeds of *Quercus serrata* Thunb (acorns). Here, studies based on NO production restriction induced by LPS in microglia cells revealed the potent inhibitory impact of triterpenoids on the mRNA expression of iNOS and COX-2 in LPS-induced BV-2 cells [123].

As mentioned previously, nitric oxide is one of the most studied promoters of neuroinflammation. The phenomenon results from the fact that localization of NOSs (NO synthases) allow for the synthesis of nitric oxide in macrophages microglia, neurons, and endothelial cells, leading to immunomodulation and neuroinflammation [124]. Lignans and neolignans are among the compounds revealing NO-inhibiting activity. These can counteract neuroinflammation and NO production by reducing the expression of PGE2, TNF-α, IL-1β, and COX2, as well as by downregulating the MAPK, ERK, and JNK pathways. Among them, the most intensively studied are balanophonin [125], chaenomiside A [126], sambucuside [127], melongenamide C, and cannabisin F [128].

The polyphenols revealed similarly high anti-neuroinflammatory and NO generation inhibitory activity. Gingerol, a compound isolated from *Zingiber officinale*, is commonly recognized as an anti-neurodegeneration agent. It is known that the polyphenol is able to inhibit NO production and pro-inflammatory cytokines via the NF-κB pathway. Additionally, other ingredients of *Zingiber officinale* (i.e., zingerone, 6-gingerol) inhibit NO production, IL-6, IL-1β, TNF-α, and mRNA levels in BV2 microglial cells activated by LPS [124]. Related activity was also observed for coumarins. Here, omphalocarpin obtained from *Toddaliae asiaticae* (Rutaceae) revealed an impact on the expression of proinflammatory mediators, such as NO, TNF-α, and IL-1β, and fostered the downregulation of COX-2 and NOS expression in LPS-stimulated BV2 cells [124,129].

## 8. The Influence of Iron Ions on Neurodegeneration Process

What are deemed ‘essential metals’ play crucial roles in the maintaining good health. Among the metals, the most important are iron, zinc, copper, chromium, and manganese. These are responsible for a number of crucial processes in our body, including enzymatic reactions (catalase, hydrogenase) and cellular activities [4]. Nevertheless, there is a thin line between the beneficial and harmful role of the elements. Numerous studies reveal the effects of excessive levels of trace metal ions with regard to mitochondrial dysfunctions, endoplasmic reticulum stress, oxidative stress, and autophagy dysregulation. One of the most important metals in this regard is iron and its ions. These have been intensively studied towards their influence on neurodegeneration processes, including AD [130].

A critical factor leading to various cellular changes is oxidative stress. The basis of free radical creation is the Haber–Weiss reaction, which is the source of the most dangerous hydroxyl radical:

$$\mathrm{Haber}–\mathrm{Weiss}\;\mathrm{reaction}:\;{O_{2}}^{•-}+H_{2}O_{2}\;\overset{{\mathrm{Fe}}^{2+}/{\mathrm{Fe}}^{3+}}{\to }{\;}^{•}\mathrm{OH}+{\mathrm{OH}}^{-}+O_{2}$$

Here, acting as catalysts, Fe^2+^/Fe^3+^ drive the reaction via the Fenton reaction, in which Fe^2+^ reacts with H_2_O_2_, leading to the generation of Fe^3+^, which is subsequently reduced with O_2_^•^^−^ [131].

Similarly to the free redox active form of iron, heme-iron plays an important role in oxidative stress generation. An overload of free heme is toxic due to their pro-oxidant activity resulting from their being part of the prosthetic group in proteins [132].

**Figure 1:**
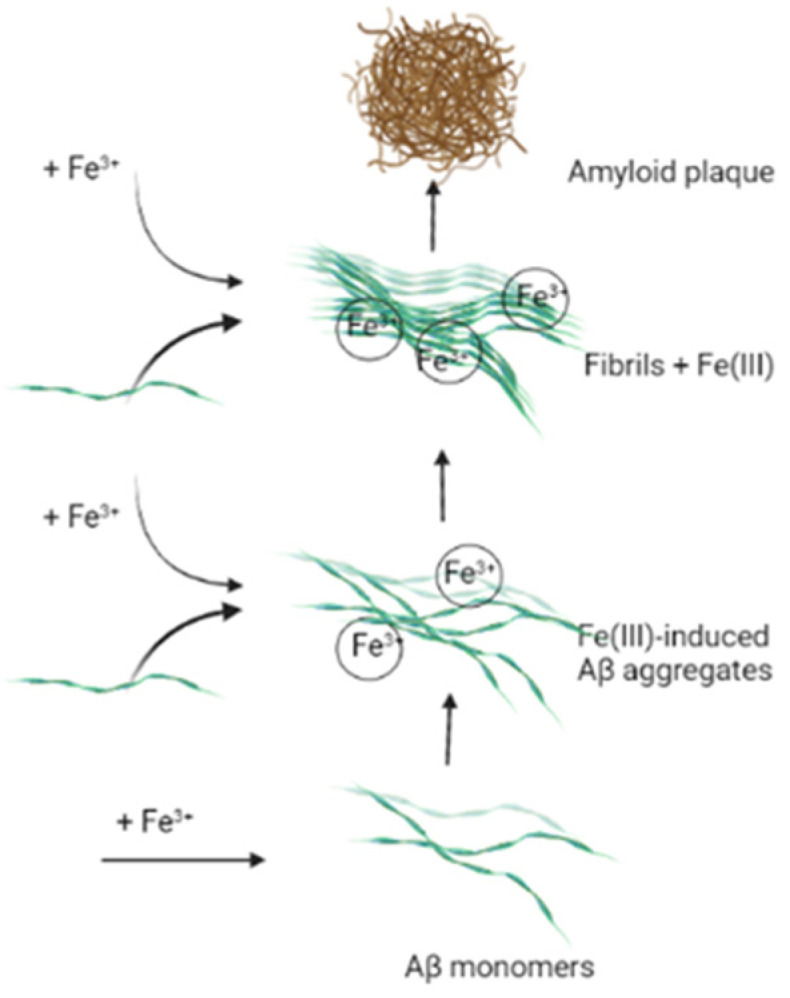
Amyloid plaques formation in Fe(III) participation.

In addition to oxidative stress involving iron ions, the metal is engaged in Aβ creation (Figure 1). Intensive studies have revealed the explicit correlation between senile plaque deposition and the level of iron ions. Detailed molecular docking simulations indicate that His6, His13, and His14 amino acid residues of amyloid β are able to interact with iron ions. An additional factor promoting the reaction of iron–Aβ interactions is the reductive brain environment, as well as the high level of metal ions in this organ [133]. The mechanism of the Aβ formation with iron ion participation can be controlled by intracellular iron via the iron regulatory element RNA stem loop in the 5ʹ unsaturated region of the APP transcript. The element was found to physiologically bind with iron response protein 1 and not with iron response protein 2 in human neuronal cells [134].

An equally significant mechanism of Aβ generation to which iron contributes is explained by furin and secretase activity, both of which are engaged in nonamyloidogenic and amyloidogenic changes in APP. Silvestri et al. revealed the explicit dependency between level of cellular iron and furin level, namely, that the protein level decreased along with an excess of the former, and simultaneously, iron content supported enhanced β-secretase activity and the development of the amyloidogenic pathway [135].

The presence of intraneuronal neurofibrillary tangles (NFTs) is an equally important factor contributing to neurodegeneration and Alzheimer’s disease. Herein, detailed and long-lasting studies have revealed a clear correlation between Fe^3+^ and the aggregation of hyperphosphorylated tau. The mechanism of the process is explained by iron metal participation in tau-tau interactions and dimerization [136]. Additionally, scientists indicated phosphorylation as an agent in metal interactions. NMR analysis confirmed strong interactions between Fe(III) and His residue of tau [137].

Proposed solutions to the negative influence of excessive levels of iron ions upon the organism include metal ion reduction (Fe^3+^) and metal ion chelation (Fe^2+^/^3+^). Both approaches are thought to allow for the keeping of an appropriate level of the metal ions in the body, while counteracting neurodegeneration development. Intense studies, both in vitro and in vivo, have revealed the ability of natural compounds to reduce and/or chelate iron ions. The most interesting and promising results are presented below.

Phenols, a rich group of secondary plant metabolites, have been intensive studied towards iron ion reduction and chelation activity. The phenomenon results from the rich biological activities of the compounds, as well as their structures. The catechol (1,2-dihydroxybenzene) nucleus, for example, has an affinity for metal ion. Moreover, the keto groups and their nearby hydroxyl groups in the flavonoids, also contribute towards iron ion reduction [138].

Studies performed for 3-hydroxyflavone, 5,7-dihydroxyflavone, and 4′-dihydroxyflavone demonstrate the positive influence of the moieties on phenol metal binding. Here, stability constant analysis reveals that 3′,4′-hydroxy substitutions at the catecholic site are most significant for ferric complexation [139].

As mentioned above, iron ions take part in ROS generation via Fenton reaction. The impact of polyphenols on the process was analyzed in terms of the participation in an inhibitory way (via formation of inert metal complexes) and in a stimulatory/pro-oxidant manner. Interesting study results have been obtained, for example, for quercetin, a well-known phenolic present in numerous fruits and vegetables. In this study, the iron-binding ability of the compound was analyzed by means of NMR and EPR spectroscopies. The resulting binding constant analysis explicitly indicated that quercetin can bind Fe(II) stronger that ferrozine, a well-known Fe(II) chelator. The researchers concluded that the high ability of quercetin to chelate Fe(II) can completely inhibit the Fenton reaction, leading to significantly improvement of oxidative stress [140].

**Figure 2:**
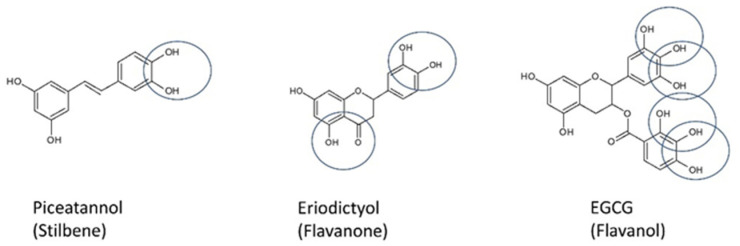
Examples of polyphenols with various numbers of metal chelator sites.

Besides catechol moiety, combinations of hydroxyl and carbonyl groups play pivotal roles. In this case, the metal binding site is defined by their assemblage. Polyphenols can be divided into the following groups: (1) ‘one-metal binding site’, having one potential chelator site—these include the curcuminoids, lignans, stilbenes, isoflavonoids, flavanols, and anthocyanins; (2) ‘two-metal binding site’, having two potential chelator sites, among others, the flavones and flavonones; (3) ‘three-metal binding site’, having three potential chelator sites—these embrace the flavonols, flavanols, and tannins; and (4) compounds having four or more metal chelator sites [141] (Figure 2).

**Figure 3:**
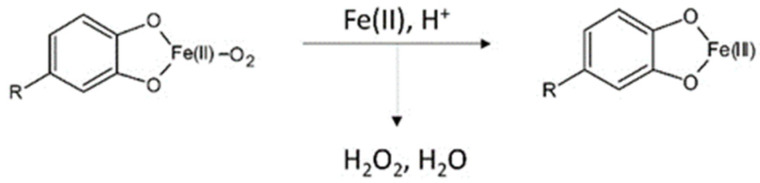
Possible mechanism reaction of Fe(II)-phenol binding reaction. The figure was prepared on the basis of [138].

A further aspect of iron ions binding by phenols is the difference in kinetic reaction in Fe(II) and Fe(III) binding. In order to expand knowledge in this direction, researchers performed studies based on gallic acid, caffeic acid, catechin, and rutin. Accordingly, affinity of the common phenols for iron ions turned out to be different. However, assessment of metal-polyphenol interactions and the redox process is difficult due to sensitivity of metal autoxidation processes and redox potentials to pH. This was overcome by performing the experiments in neutral phosphate buffer. The outcome of the work established that (1) spectral changes following Fe(II) addition are much faster and more intense than with Fe(III); (2) Fe(II)-polyphenol binding does not provide protection to Fe(II) against autoxidation; and (3) Fe(II)-polyphenol binding is faster than autoxidation of free Fe(II) [138]. Conclusions drawn on the basis of the conducted research indicate that the common polyphenols can bind Fe(II) and Fe(III), but the second is captured more slowly. The mechanism of the reaction is explained by electrons transfer from Fe(II) with the concomitant formation of Fe(III)-phenol complexes (Figure 3). The reaction could also take place starting from Fe(III)-phenol complexes; however, the slow preliminary step of Fe(III) reduction by phenols hampers it.

Another important and well-known phenol is chlorogenic acid. Similarly to other secondary plant metabolites, the compound reveals a variety of biological activities. Studies were performed to ascertain the ability of the phenol to chelate Fe(II), to determine hydroxyl radical generation as influenced by the compound during iron release, and to understand the impact of chlorogenic acid on iron-involved polymerization. The outcome of such work revealed that chlorogenic acid can interact with Fe(III) to form complexes that can interact with ferritin via hydrogen bonds. Additionally, the generation of hydroxyl radicals is significantly reduced by the phenol during ion release. Moreover, chlorogenic acid can also promote the rates of ion oxidative deposition and ion release from ferritin [142].

Reduction or chelation of iron ions by natural compounds such as phenols was confirmed repeatedly in studies based on essential oils rich in these compounds. In most of these studies, correlation between high ability of essential oils to bind iron ions and content of phenols, terpenes, or other secondary plant metabolites indicate that the high biological activity can be related to the main components of the essential oils [143].

Activity of secondary plant metabolites towards regulating organism levels of iron ions by their reduction and chelation was confirmed in in vivo studies using rat models. Both an acute and a long-term study using rat models have indicated the significant influence of the polyphenol on iron level. In the acute study, duodenal mucosa to quercetin was found to increase apical iron intake and to decrease subsequent basolateral iron efflux in the circulation. The probable mechanism of action is thought to be based on the chelation ability of quercetin in binding iron between 3-hydroxyl and 4-carbonyl groups and by methylation of the 3-hydroxyl group. In the case of the long-term study, assessment of the positive influence of quercetin was based on recapitulation in Caco-2 cells exposed to quercetin. Here, reporter assays in Caco-2 cell suggested that the repression of FPN by quercetin was not a transcriptional event but might be mediated by miRNA interaction with the FPN 3′UTR [144].

Another natural compound revealing excellent biological activity is tannic acid. Besides reduction and chelation activity in vitro, the compound has revealed an ability to counteract iron overload by its chelator activity. The obtained research results were similar to that of the control substance (desirox-a standard iron chelator). Additionally, it is probable that tannic acid might modulate DMT-1, block L-type calcium channels, and reverse iron overload in the organism [145].

Epigallocatechin-3-gallate (EGCG) is a similarly important and well-known natural compound. Detailed analysis has demonstrated the ability of EGCG to decrease cellular assimilation of heme iron, along with the ability to limit its basolateral efflux. The data confirmed that the polyphenol constrains heme iron absorption by reducing basolateral iron exit, rather than by decreasing apical heme iron uptake in intestinal cells [146].

## 9. Conclusions

Although not fully understood, the pathological processes associated with AD are influenced by many factors. Many in vitro and in vivo studies have demonstrated that natural plant products and phytochemicals exhibit neuroprotective effects. Among the many mechanisms are activity targeting cholinergic neurotransmission and neuroinflammation; generating an imbalance of iron in the organism; inhibiting α-synuclein, as well as BACE and MAO; affecting Aβ accumulation and aggregation; and reducing tau phosphorylation, as well as inducing anti-inflammatory and antioxidant effects.

The plant metabolites and their combinations are a valuable collection of natural products that should be tested to prevent and effective treat AD. These naturally based drugs will surely have fewer side effects than the currently available pharmacological treatments.

## Figures and Tables

**Table 1:** Plant products with anti-AD potential possess activity targeting cholinergic neurotransmission.

Plant	Extract	Model and Assay	Target	Results	Ref.
*Salvia triloba* L.	aerial parts macerated in 70% methanol	AD rats, male (administration of AlCl_3_)	AChE, CRP, NF-κB, MCP-1	↓AChE activities in brain and serum, ↓CRP, ↓NF-κB, ↓MCP-1, ↑ACh	[19]
*Salvia triloba*	samples extracted with 75% ethanol at r.t.	in vitro enzymatic assay (AChE), Swiss albino mice, male scopolamine-induced amnesia	AChE	AChE inhibition, IC_50_: 0.71 mg/mL, memory-enhancing effect: 57.1 and 71.4% at 200 and 400 mg/kg, respectively	[20]
*Melissa officinalis*	samples extracted with 75% ethanol at r.t.	in vitro enzymatic assay (AChE), Swiss albino mice, male scopolamine-induced amnesia	AChE	↓AChE activities in brain, memory-enhancing effect: no significance	[20]
*Teucrium polium*	samples extracted with 75% ethanol at r.t.	in vitro enzymatic assay (AChE), Swiss albino mice, male scopolamine-induced amnesia	AChE,	AChE inhibition, IC_50_: 0.55 mg/mL, memory-enhancing effect: 55.4 and 61.6% at 200 and 400 mg/kg, respectively	[20]
*Piper nigrum*	seeds extracted with 70% methanol at r.t.	SD rats, male (administration of AlCl3)	AChE, CRP, NF-κB, MCP-1	↓AChE activities in brain and serum, ↓CRP, ↓NF-κB, ↓MCP-1, ↑ACh	[19]
*Foeniculum vulgare*	fruit extracted with 90% methanol at r.t.	Swiss mice, scopolamine- and aging-induced amnesia	AChE	amnesia behavioral improvement, ↓AChE activities↓ in brain	[21]
*Ocimum sanctum*	water extract, refluxed at 75–80 °C	Wistar rats; male; maximal electroshock-, atropine-, and cyclosporine-induced dementia	AChE	cognitive behavioral performance improvement	[22]
*Ocimum sanctum* Linn	leaf extracted with 95% ethanol extract using Soxhlet	Wistar rats; male; maximal electroshock-, atropine-, and cyclosporine-induced dementia	AChE	cognitive behavioral performance improvement ↓AChE activities↓ in cortex, cerebellum, medulla oblongata, and midbrain region: 21%, 21%, 25%, and 30% at 500 mg/kg	[22]
*Lavandula angustifolia* Mill.	essential oils obtained from steam distillation	C57BL/6J mice, male, scopolamine-induced amnesia; H_2_O_2_-induced PC12 (1.5–50 μg/mL LO for 24 h)	AChE, ROS, MMP	cognitive behavioral performance improvement, ↓AChE activities, ↓MDA, ↑SOD activities↑, ↑GPX activities, PC12 cells model: ↓LDH, ↓NO, ↓ROS, ↑MMP	[23]
Olive oil (fruit oil of *Olea europaea*)	olive oil (rich in oleic acid)	ICR mice, male, intracerebroventricular injection of Aβ into the mice brain	MDA, NO, COX-2	↓MDA, ↓NO, ↓COX-2	[24]
Corn oil (*Zea mays*)	corn oil (rich in linoleic acid)	ICR mice, male, intracerebroventricular injection of Aβ into the mouse brain	AChE, MDA, NO, iNOS, COX-2	↓AChE, ↓MDA, ↓NO, ↓COX-2, ↓iNOS	[24]
Perilla oil (*Perilla frutescens*)	perilla oil (rich in α-linolenic acid)	ICR mice; male, intracerebroventricular injection of Aβ into the mouse brain	AChE, MDA, NO, iNOS, COX-2, BDNF	↓AChE, ↓MDA, ↓NO, ↓COX-2, ↓iNOS↓, ↑BDNF	[24]
Coffee	boiled water extraction	in vitro enzymatic assay	AChE	AChE inhibition, IC_50_: 0.41 ± 0.004 mg/mL	[25]
Shaddock (*Citrus maxima*)	citrus fruit juices	Fe^2+^-induced malondialdehyde production in rat brain homogenate in vitro	AChE	AChE inhibitory rate of 60.39% at 66.68 mL/L and of ≈ 28% at 16.67 mL/L	[26]
Grapefruit (*Citrus paradisii*)	citrus fruit juices	Fe^2+^-induced malondialdehyde production in rat brain homogenate in vitro	AChE	AChE inhibitory rate of ≈ 52% at 66.68 mL/L; AChE inhibitory rate of ≈ 29% at 16.67 mL/L	[26]
Lemon (*Citrus limoni*)	citrus fruit juices	Fe^2+^-induced malondialdehyde production in rat brain homogenate in vitro	AChE	AChE inhibitory rate of ≈ 48% at 66.68 mL/L and of ≈ 22% at 16.67 mL/L	[26]
Orange (*Citrus sinensis*)	citrus fruit juices	Fe^2+^-induced malondialdehyde production in rat brain homogenate in vitro	AChE	AChE inhibitory rate of ≈ 50% at 66.68 mL/L and of ≈ 30.89% at 16.67 mL/L	[26]
Tangerine (*Citrus reticulata*)	citrus fruit juices	Fe^2+^-induced malondialdehyde production in rat brain homogenate in vitro	AChE	AChE inhibitory rate of ≈ 57% at 66.68 mL/L and of ≈ 20% at 16.67 mL/L	[26]
Extra-virgin olive oil (*Olea europaea*)	extra-virgin olive oil	TgSwDI model	Aβ, tau, ApoE, PPARγ, and LXRs	cognitive behavioral performance improvement, ↓Aβ, ↓tau, ↓phosphorylation of tau, ↑ApoE, ↑PPARγ, ↑LXRs ↑Aβ clearance pathways	[27]
Green tea (*Camellia sinensis*)	water extract of green tea	in vitro enzymatic assay	AChE, BuChE, and BACE-1	AChE inhibition, IC_50_: 7.2 μg/mL	[28]
White tea (*Camellia sinensis*, WTE)	water extract of white tea	in vitro enzymatic assay	AChE	AChE inhibition, IC_50_: 8.06 μg/mL	[28]
Green tea (*Camellia sinensis*, GTE-PG)	water extract of green tea processed through simulated gastrointestinal digestion to obtain post-gastric digested extract	in vitro enzymatic assay	AChE	AChE inhibition, IC_50_: 17.84 μg/mL	[28]
Green tea (*Camellia sinensis*, GTE-CA)	water extract of green tea processed through simulated gastrointestinal digestion to obtain colon-available digested extract	in vitro enzymatic assay	AChE	AChE inhibition, IC_50_: 9.59 μg/mL	[28]
White tea (*Camellia sinensis*, WTE-PG)	water extract of white tea processed through simulated gastrointestinal digestion to obtain post-gastric digested extract	in vitro enzymatic assay	AChE	AChE inhibition, IC_50_: 16.1 μg/mL	[28]
White tea (*Camellia sinensis*, WTE-CA)	water extract of white tea processed through simulated gastrointestinal digestion to obtain colon-available digested extract	in vitro enzymatic assay	AChE	AChE inhibition, IC_50_: 4.22 μg/mL	[28]
Black tea (*Camellia sinensis*)	water extract of black tea	in vitro enzymatic assay	AChE and BuChE	AChE inhibition, IC_50_: 0.06 ± 0.005 mg/mL; BuChE inhibition, IC_50_: 0.05 ± 0.007 mg/mL	[25]
Green tea (*Camellia sinensis*)	water extract of green tea	Wistar rats; male; injection with green tea extract, saline, or AlCl_3_ into the left-brain hemisphere cornu ammonis region 1 of the hippocampus	AChE	↑COX and AChE activities with GTE injection, ↓AlCl_3_ neurotoxicity, 3-epigallocatechin gallate and epicatechin in extract improves cholinergic synaptic functions	[29]
Black tea (*Camellia sinensis*)	brewed at 85 °C	Wistar rats, male, AlCl_3_ (100 mg/kg, i.p. 60 days) induced AD	AChE, APP, β and γ secretases, Aβ	memory-enhancing effect ↓TBARS, ↑GSH, ↑SOD, ↑catalase, ↑GPx	[30]

**Table 2:** Selected natural compounds which activity towards BACE-1 inhibition was confirmed in in vitro, in silico, or in vivo studies.

In Vitro and In Silico Studies towards BACE-1 Inhibition				
Compound	Type of Study/Methodology	Mechanism of Action	Studies Results/Comment	References
Two serratene-type triterpenoids: lycernuic acid A with a ρ-hydroxycinnamate group as an ester substituent and 21β-hydroxyserrat-14-en-3,16-dione extracted from *Lycopodiella cernua* L.	BACE1 fluorescence resonance energy transfer (FRET) assay kit Molecular docking simulation in ChE inhibition-Autodock VINA	Interactions with several pocket domains of the AChE, which were 5 Å from the inhibitors in the original complex.	IC_50_ = 0.23 μM and 0.98 μM, respectively. The compounds revealed higher inhibitory activity than quercetin, a positive control.	[31,45]
Embelin (3-undecyl-1,4-benzoquinone) from *Embelia ribes*	The BACE-1 fluorescence resonance energy transfer (FRET) assay kit Molecular modelling using Maestro v9.0 and Impact program v5.5	Molecular docking revealed entering of embelin into the active site gorge and interacting with Tyr71 (via hydrogen bonding).	IC_50_ = 2.11 μM. Lower activity than donepezil, a positive control.	[31,46]
Five arylbenzofurans: sanggenofuran A, mulberrofuran D, mulberrofuran H, morusalfuran B, and mulberrofuran D2 from the root bark of *Morus alba*	BACE1 fluorescence resonance energy transfer (FRET) assay kit Molecular docking analysis carried out in AutoDock 4.2. but only for one compound, mulberrofuran D2	Molecular docking revealed the following interactions for mulberrofuran: D2 bound to the active allosteric site of BACE-1 through hydrogen bonds with Asn37, Ser36, and Tyr198, as well as hydrophobic interactions with Val69, Tyr71, Trp76, Phe108, Tyr198, and Ile126	Sanggenofuran A revealed lower activity (IC_50_ = 5.64 μM) than mulberrofuran D (IC_50_ = 3.74 μM), and both compounds were less active than quercetin (IC_50_= 3.38 μM). The remaining compounds revealed higher activity in comparison to quercetin: mulberrofuran D2, mulberrofuran H, and morusalfuran B, for which IC_50_ was equal to: 0.73 μM, 1.04 μM, and 2.03 μM, respectively.	[31,47]
Fifteen ptesorin derivatives from *Pteridium aquilinum*	BACE1 FRET assay Docking studies carried out using AutoDock 4.2.6 software	(2R)-Pteroside D was able to bind (hydrogen bonds) with Asn37, Trp76, and Ile126; (2R,3R)-pteroside C was able to create hydrogen bonds with Ser36, Asn37, Asp228, and Thr231, as well as hydrophobic interactions with Ala39, Trp76, Val69, Ile118, and Arg129; (3S)-pteroside D was able to create hydrogen bonds with Ser36, Asn37, Ile126, and Asp228, as well as hydrophobic interactions with Val69, Tyr71, Trp76, and Arg128.	The most active compounds were the following: (2R)-pteroside D, (2S,3R)-pteroside C, (2R,3R)-pteroside C, and (3S)-pteroside D (IC_50_ = 2.55, 9.17, 3.77, and 27.4 μM, respectively). (2R)-Pteroside D, (2R,3R)-pteroside C, and (3S)-pteroside D revealed higher inhibitory activity than quercetin. The compounds revealed the ability to bind with crucial amino acid residues, creating BACE-1 binding sites.	[48]
Three phlorotannins: eckol, dieckol, and 8,8′-bieckol isolated from *Ecklonia cava*	Fluorometric assays with recombinant human BACE1 Molecular docking with the use of Autodock Vina software version 1.1.2	Dieckol revealed the ability to interact with Trp76, Thr232, and Lys321 through hydrogen bonds. 8,8′-Bieckol interacted with the BACE-1 active site by hydrogen bonding interactions with Lys107, Gly230, Thr231, and Ser325.	Dieckol and 8,8′-bieckol revealed higher inhibitory activity than reseveratrol (positive control) with IC_50_ = 2.34 and 1.62 μM, respectively.	[49]
Flavonoids and non-flavonoids: caffeic acid, hydroxytyrosol, oleuropein, verbascoside, quercetin, rutin, and luteloin isolated from *Olea europaea* L.	BACE inhibitor screening assay kit	The compound structure analysis suggests that the 3,4-dihydroxy group and double bond in olive biophenols can interfere with hydrogen bonds of the NH_2_ group and NH hydrogens in the core structure of the BACE-1 enzyme. The higher activity of flavonoid olive biophenols in comparison to non-flavonoid olive biophenols results from their chemistry-a 15-carbon skeleton consisting of two benzene rings linked via the heterocyclic pyrene ring-C.	Caffeic acid, hydroxytyrosol, oleuropein, verbascoside, quercetin, rutin, and luteloin revealed higher inhibitory activity than positive control epigallocatechin gallate, with the following IC_50_ values: 16.67, 0.035, 2.76, 0.0063, 0.55, 0.0038, and 0.52 μM, respectively.	[50]
Flavonoids: bavachin, bavachinin, bavachalcone, and iso-bavalchacone isolated from *Psoralea fructus*	BACE-1 activity assay performed using assay kits Docking studies conducted using Autodock Vina	Structure analysis of studied compounds revealed that the chalcone backbone of bavachalcone and isobavachalcone was more flexible, which allowed them to fit more easily to the conformations of Aβ42 and enabled more hydrogen bonds than the flavanone of bavachin and bavachinin. Bavachalcone and isobavachalcone may stabilize Aβ42 monomers through their strong bindings, whereas bavachinin might induce intricate conformational changes of Aβ42 through binding, which leads to the off-pathway aggregation.	BACE-1 inhibition: 14% (bavachin at concentration 100 μM), 20% (bavachinin at concentration 100 μM), 68% (bavalchacone at concentration 100 μM), and 34% (iso-bavalchacone at concentration of 100 μM).	[51]
Linalool and 2,3,4,4-tetramethyl-5-methylene-cyclopent-2-enone isolated from *Lavandula luisieri*	proBACE-1 enzymatic assay	Lack of mechanisms analysis.	Inhibitory activity for linalool was equal to 4.7, whereas 2,3,4,4-tetramethyl-5-methylene-cyclopent-2-enone was 31.8% at a concentration of 45 μg/mL.	[52]
Ajmalicine and reserpine	Molecular docking with use of AutoDock 4.2 BACE-1 inhibitory assay	Strong binding of the compounds to the catalytic site of BACE-1. Reserpine interacted with Thr72, Asp32, and Asp217 by five hydrogen bonds, whereas ajmalicine was able to create hydrophobic interactions with Asp32 and Asp228. Thanks to the reserpine indole ring, the compound acted as a hydrogen bond donor capable of creating double hydrogen bonds with the catalytic site of the enzyme, whereas ajmalicine bound more strongly to the enzyme by hydrophobic interactions.	AJM showed the maximum inhibition of BACE-1 activity to be 69% at 50 μM concentration, whereas RES imparted 47% inhibition at the same concentration.	[53]
(S)-5,7,3′,5′-Tetrahydroxy-flavanone-7-O-(6″-galloyl)-β-D-glucopyranose **(1)**; flavanone: (S)-5,7,3′,5′- tetrahydroxy-flavanone-7-O-β-D-glucopyranose **(2)**, and dihydrochalcones: 4,2′,6′-trihydroxy-dihydrochalcone-4′-O-(6″-galloyl)-β-D-glucopyranose **(3)**; 3,4,2′,6′-tetrahydroxydihydroflavone- 4′-O-β-D-glucopyranose **(4)**; 3,4,2′,6′-tetrahydroxy- dihydrochalcone-4′-O-(6″-galloyl)-β-D-glucopyranose **(5)**; and phloretin 4′-O-[4′, 6′-O-(S)-HHDP]-β-D-glucoside **(6)** isolated from *Balanophora* *involucrata Hook.*	Fluorescent resonance energy transfer (FRET) peptide cleavage assay	Lack of mechanism analysis.	In vitro studies revealed the activity of the compounds to inhibit BACE-1; nevertheless, only compounds **1**, **2**, **4**, and **5** turned out to be a little more active than the positive control. None of the substances achieved an inhibition capacity of 50% at 10 μM concentration.	[54]
The chemical components of *W. fruticosa viz.* botulin, betulinic acid, ursolic acid, ellagic acid, quercetin, kaempferol, oenothein C, and cyanidn-3,5-diglucoside.	Fluorescence resonance energy transfer (FRET) assay Molecular docking with the use of Schrodinger’s Glide Module	The high activity of ellagic acid resulted from hydrogen bonding with Thr231, Asp228, Gly34, and Trp76 amino acid residues. Additionally, hydrophobic interactions were observed between aromatic rings of the acid and Trp115 and Tyr71 residues.	Among the compounds, ellagic acid and quercetin revealed the highest activity (70% BACE-1 inhibition at 100 μM). The most active was ellagic acid (IC_50_ = 16.2 μM).	[55]
3,4-di-*o*-Caffeylquinic acid, apigenin, and 7-*o*-methylwoonin isolated from *A. paniculata*	Molecular docking with the use of the Glide tool BACE-1FRET assay kit	The 3,4-di-*o*-caffeylquinic acid was able to bind with Trp71, Phe108, Gly34, Arg128 (first pose) and Ile126, Trp76, and Tyr198 (second pose) by hydrogen bonds. Hydrophobic interactions were also observed.	BACE-1 inhibition assay indicates that 3,4-di-*o*-caffeylquinic acid is the most promising inhibitor (activity slightly higher than quercetin), whereas the activity of 7-o-methylwogonin was similar to quercetin and the activity of apigenin was slightly weaker than quercetin. In accordance with molecular docking, 3,4-di-*o*-caffeylquinic acid showed the highest ability to bind with the BACE-1 active site. Hydrophobic interactions and hydrogen bonds allow achieving selective BACE-1 inhibition by the compound.	[56]
Proroberberine alkaloids: berberine, palmatine, jateorrhizine, epiberberine, coptisine, groenlandicine, and eporphine alkaloid-magnoflorine from *Coptidis Rhizoma*	BACE-1 inhibitory assay based on manufacturer protocol	The activity of epiberberine and groenlandicine is closely related with the presence of the methylenedioxy group in the D ring that is responsible for the BACE-1 inhibitory activity of protoberberine alkaloids.	Among the compounds, only epiberberine and groenlandicine revealed good, non-competitive BACE-1 inhibitory activities, with IC_50_ = 8.55 and 19.68 μM, respectively.	[57]
**In Vivo and Ex Vivo Studies towards BACE-1 Inhibition**				
**Compound**	**Animal Models/Type of Study/Methodology**	**Mechanism of Action**	**Studies Results/Comment**	**References**
Berberine (isoquinoline alkaloid)	New Zealand white rabbits. Lesion (pro-Alzheimer’s disease) was induced by aluminum-maltol injection into intraventricular fissure. Berberine chloride (50 mg/kg) was administered intragastrically once daily for 14 days. Histopatological examinations (brain tissue) were performed. BACE-1 activity was detectable by RP-HPLC.	The mechanism of CNS cell damage prevention by berberine was based on BACE-1 inhibition, as well as its antioxidant, anti-inflammatory, and AChE inhibitory activities.	Results indicated that berberine chloride has a preventative effect on the degeneration of the hippocampus, along with the ability to decrease the activity of BACE-1. Berberine prevented the increase in enzyme activity in 40% of all cases, as compared with the control group.	[58]
2,2′,4′-Trihydroxychalcone (TDC) from *Glycyrrhiza glabra*	APP-PS1 double transgenic mice model (B6C3-Tg (APPswe, PS1dE9)). The studied substance was administered i.p. by 100 days to two groups with different doses (9 mg/kg/day and 3 mg/kg/day). The mice were applied to the MWM spatial memory test. Additionally, Western blot analysis for BACE-1 was conducted.	This is a specific non-competitive BACE-1 inhibitor. Taking into account the low molecular weight of TDC, it is highly probable that the compound is able to cross the blood–brain barrier in vivo.	Administration of TDC (9 mg/kg/day) caused significant decreasing of Aβ production and senile plaque formation. The activity resulted in memory improvement, as observed in the Morris water maze test. It was also determined that the level of BACE-1 in TDC-treated Tg mice was almost kept unchanged, as compared with those in the vehicle-treated Tg mice.	[59]
Gallic acid	Male B6.Cg-Tg(APPswe, PSEN1dE9) 85Dbo/Mmjax mice (bearing ‘Swedish’ APPK595N/M596L and PS1 exon 9-deleted mutant human transgenes) on a congenic C57BL/6J background (designated APP/PS1 mice). GA was administered with 20 mg/kg/day for 6 months. Two behavioral tests were conducted: Y-maze and RAWM.	The activity of gallic acid towards BACE-1 inhibition led to nonamyloidogenic APP metabolic effects. GA is able to inhibit the enzyme activity post-translationally.	Gallic acid demonstrated the ability to mitigate impaired learning and memory and reduce cerebral amyloidosis. A 6 month oral therapy based on GA completely remediated behavioral deficits, ameliorated cerebral amyloidosis, and reduced amyloid abundance.	[60]
Anatabine	Measurement of BACE-1 expression by RT-qPCR according to SHSY-5Y cells. Pharmacokinetic studies of anatabine were performed using 43-week-old B6/SJL F1 mice. The studied substance was administered i.p. at dosages of 0.5 and 2.0 mg/kg/day over 4 days.	Mechanism of Aβ reduction was based on the impact of anatabine on BACE-1 transcription. The compound was able to reduce BACE-1 protein levels in human neuronal-like SHSY-5Y cells.	Reduction was indicated of two forms of amyloid (soluble-40% reduction and insoluble-30% inhibition) after 4 days of drug administration at a dosage of 2 mg/kg.	[61]

## Data Availability

Not applicable.

## Abbreviations

Ach	acetylcholine
AChE	acetylcholinesterase
AME	alternariol monomethyl ether
Apo E	apolipoprotein E
APP	amyloid precursor protein
BACE	β-secretase
BDNF	brain-derived neurotrophic factor
BuChE	butyrylcholinesterase
ChAT	choline O-acetyltransferase
CDK5	cyclin-dependent kinase 5
COX	cyclooxygenase
CRP	C-reactive protein
EGCG	epigallocatechin-3-gallate
GPx	glutathione peroxidase
GSH	glutathione
GSK	glycogen synthase kinase
iNOS	inducible nitric oxide synthase
LDH	lactate dehydrogenase
LXR	liver X receptor
MAO	monoamine oxidase
MDA	malondialdehyde
MCP-1	monocyte chemoattractant protein-1
MMP	mitochondrial membrane potential
NF-κB	nuclear factor kappa-B
NGF	nerve growth factor
NO	nitric oxide
PI3K	phosphatidylinositol 3-kinase
PKB	protein kinase B
PPAR-γ	peroxisome proliferator-activated receptor gamma
PP2A	protein phosphatase 2A
ROS	reactive oxygen species
r.t.	room temperature
SOD	superoxide dismutase
TBARS	thiobarbituric acid reactive substances
